# Sexual behaviour, contraceptive knowledge and use among female undergraduates’ students of Muhimbili and Dar es Salaam Universities, Tanzania: a cross-sectional study

**DOI:** 10.1186/1472-6874-14-94

**Published:** 2014-08-07

**Authors:** Magreat J Somba, Milline Mbonile, Joseph Obure, Michael J Mahande

**Affiliations:** 1 Muhimbili University College of Health and Allied Science & -Harvard Collaborative Projects, Dar es Salaam, Tanzania; 2 Department of Geography Dept, University of Dar es Salaam, Dar es Salaam, Tanzania; 3 Department of Epidemiology & Biostatistics, Kilimanjaro Christian Medical University College, P.O. Box 1638, Moshi, Tanzania; 4 Department of Obstetrics and Gynaecology, Kilimanjaro Christian Medical University College, Moshi, Tanzania

**Keywords:** Sexual behaviour, Contraceptive Knowledge, Use, University students Tanzania

## Abstract

**Background:**

The rate of premarital sexual activity, unwanted pregnancies and illegal abortions remain higher among university students. This calls for understanding the knowledge on contraceptive use and sexual behaviours among this high risk group if the incidence of unintended pregnancy, illegal abortions and high sexual risky behaviour are to be minimized. This study aimed to assess ssexual behaviour, contraceptive knowledge and use among female undergraduates’ students of Muhimbili and Dar es Salaam Universities in Tanzania.

**Methods:**

A cross-sectional analytic study was conducted among undergraduate female students in the two Universities located in Dar es Salaam region, Tanzania. The study period was from June 2013 to October 2013. A self-administered questionnaire was given to 281 students. Of these, 253 were retrieved, giving a response rate of 90%. Data was analyzed using Statistical Package for Social Science (SPSS) for Windows version 17.0. Descriptive statistics were summarized. The chi square test was used to examine relationship between various sociodemographic and sexual behaviours variables with contraceptive use. A P-value of less than 0.05 was considered statistically significant.

**Results:**

Results showed that majority (70.4%) of the students have had sexual intercourse. All participants had knowledge of contraception. More than half, 148 (58.5%) of sexually active women reported ever used contraception before while 105 (41.5%) were current contraceptive users. Majority (74.7%) of the sexually active group started sexual activity at young age (19–24 years). Condom, 221(24.3%) and pills, 153 (16.8%) were the known contraceptive methods. The most popular method of contraception used were condoms, withdrawal and periodic abstinence. The main sources of information about contraception were from friends, radio and school (39.5%, 36% and 24%) respectively. Forty (15.8%) women had pregnancies. Of these, 11 (27%) have had unwanted pregnancies among which 54.6% have had induced abortion. Marital status, age at first sex, ever had sex, ever been pregnant and unwanted pregnancies were associated with use of contraception.

**Conclusions:**

Most of the student’s had knowledge of contraception. However, rate of contraception use is still low. Majority of the respondent were sexually active, with the majority started sexual activity at young age. This needs advocacy for adolescence reproductive health education to promote the use of the available contraceptive services amongst university students.

## Background

Adolescent sexual behaviour has been recognized as an important health, social and demographic concern in the developing world [1]. Adolescent pregnancy is associated with adverse maternal, fetal and neonatal outcomes [2,3]. Teenage girls who get pregnancy suffer from social and economic consequence and they are more likely to drop out of school. Furthermore, unwanted pregnancy poses a big problem among young adult in developing countries [4].

Majority of students who join universities in Tanzania are aged between 19 and 29 years [5]. Most of female students are enrolled to university at their young age, this expose them to unplanned and unprotected sexual intercourse leading to unintended pregnancies, abortions and sexual transmitted infections [6]. The increased sexual risky behaviours of female University student has been attributed to movement from a restricted rural to a more liberal urban environment, age and marital status [7].

A recent study in Tanzania reported a 34.4% contraceptive prevalence rate among women of reproductive age (15–49 years) [8]. Results from Demographic and Health Survey in Tanzania also reported contraceptive prevalence use rate of 19% among female aged between 20 and 24 years, and the teenager pregnancy rate of 44% [9]. Previous studies conducted in sub Saharan Africa have reported on risky sexual behaviours among African youths, particularly University students. For example, a study among university students in Madagascar by Rahamefy and colleagues revealed that 29% of the students reported to have 2 or more sexual partners and only 13.5% were consistently using the condom [10]. However, a slightly higher proportion (48.9%) of condom use was reported among university students in Kampala [11].

Low level of utilization of contraceptives has been associated with high rates of unwanted pregnancies and unsafe abortions among Sub-Saharan Africa youth [12]. A study conducted among nursing female students at Calabar University in Nigeria revealed that 55% of students who were sexually active had knowledge of family planning especially condom use (37%) [13]. In a similar study, more than half (51%) of the students who had unwanted pregnancy ended to abortion [13]. Low utilization of contraception has also been attributed to limited capacity of the health care system and structure within which family planning services are offered [14]. Furthermore, individual factors such as risk perception, fear of side effects, opposition from male partners, health service limitations and insufficient knowledge needed to make informed choices have been reported as barriers for utilization of contraception [15,16].

As an attempt to curb the problem, Tanzania government policy on family planning has made an effort to ensure the availability of contraceptives services in its health Centres for men and women who are ready for and in need [17]. In line with the government policy, the University of Dar es Salaam and Muhimbili established a project to target students and other youths in the surrounding communities by providing reproductive health services including contraceptives. However, the effectiveness of this program has not yet evaluated.

Previous studies in sub Saharan Africa have demonstrated that University female students are at high risk of sexual transmitted infections including HIV, and they have high rate of unwanted pregnancy which results to high abortion rate. Despite this fact, there is limited information about sexual behaviours, contraceptive knowledge and use among female University students in Tanzania. This underscores the need to understand the sexual risk behaviour, knowledge and pattern of contraceptives use among this high risk group in order to promote proper use of contraception. Therefore this study aimed to examine sexual behaviour, contraceptive knowledge and use among female undergraduates’ students of Muhimbili and Dar es Salaam Universities in Tanzania. The study also explored factors associated with use of contraception.

## Methods

### Study design and setting

**Figure 1:**
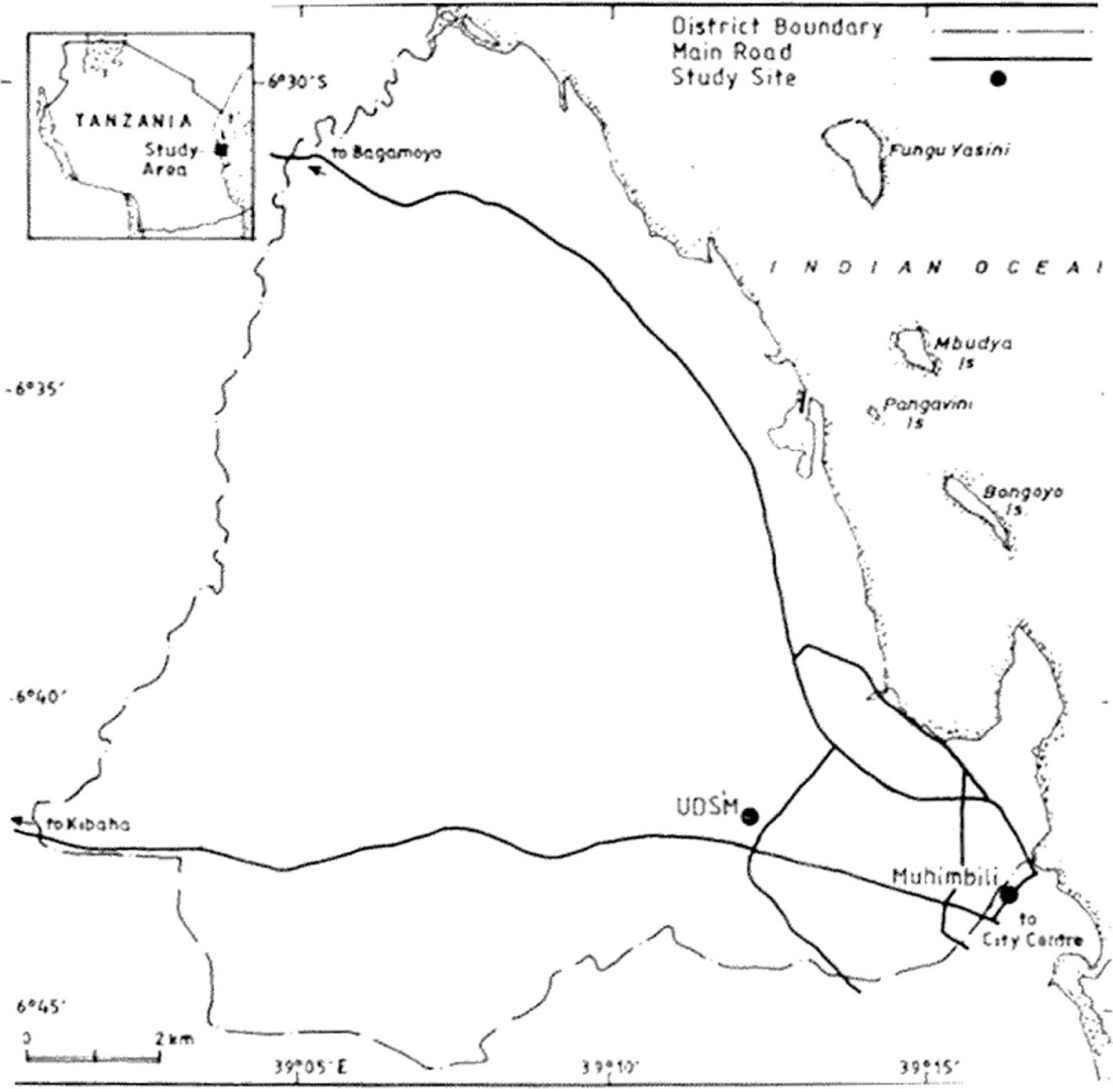
Location of the Study Area in Dar es Salaam Region.

A cross-sectional analytic study was carried out from June 2013 to October 2013 in Dar es Salaam region of Tanzania. Two universities, one medical University (Muhimbili University of Health and Allied Science i.e. MUHAS) and University of Dar es Salaam (UDSM)) which is a non-medical were conveniently selected (Figure 1). From each participating institution, we only recruited women undergraduate students (married and unmarried).

For MUHAS, students were selected according to the year of study (i.e. year one to year 5), where the total numbers of students selected for each year was proportion to size of respective year of study. We used similar selection criteria (proportion based to size) for the UDSM, except that a study program instead of year of study was used because the UDSM comprised of more than on study programs (i.e. science or arts and humanity). The female students were selected based on the fact that high proportion of unmarried university students in sub-Saharan Africa have been reported to engage in unprotected sexual relations which has resulted to an increased rate of abortion and unwanted pregnancies [18,19].

**Figure 2:**
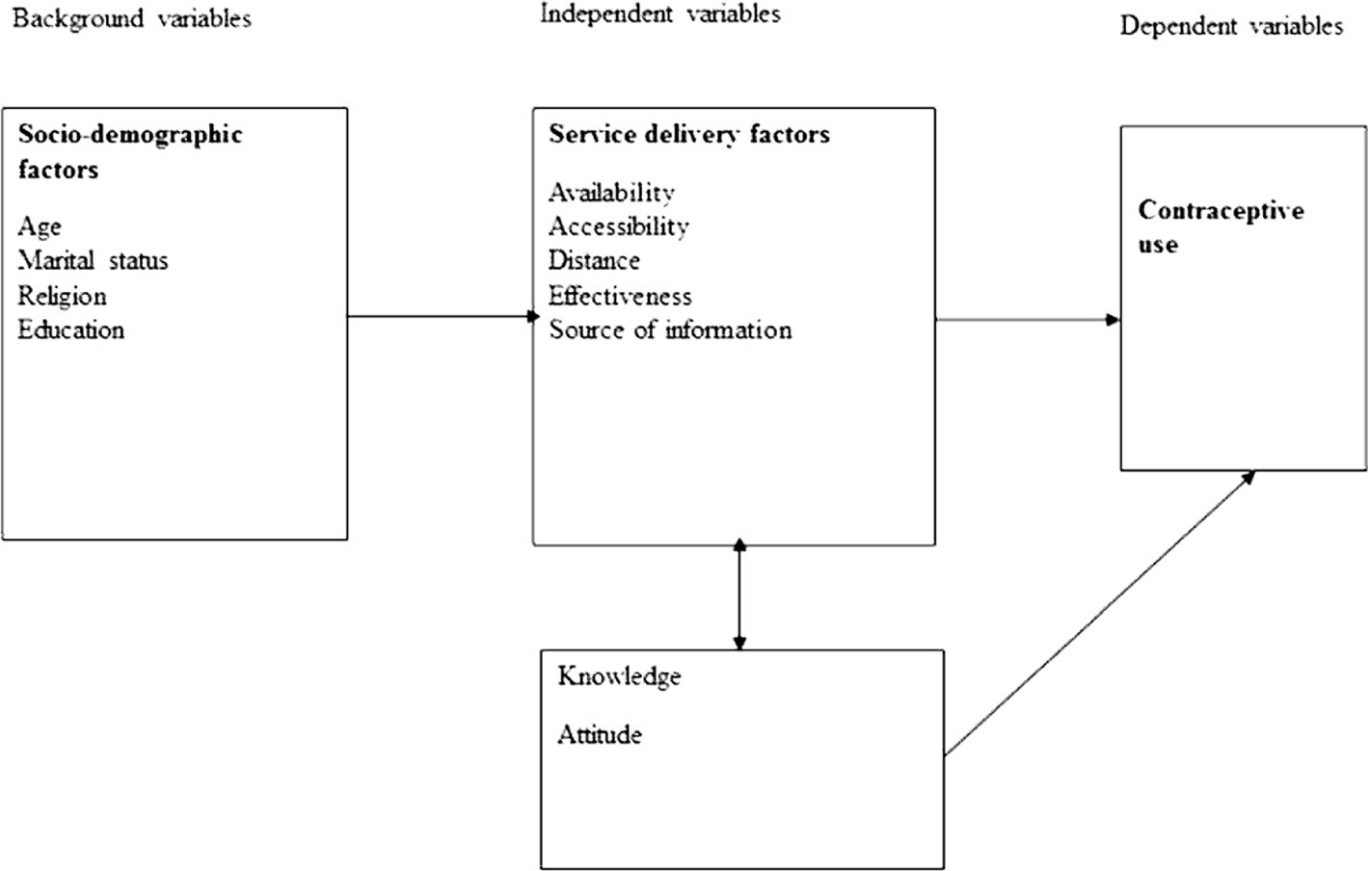
A simplified model showing the variables that influences contraceptive use.

Dar es Salaam University and Muhimbili University of Health and Allied science (MUHAS) are the oldest universities in Tanzania. Both Universities are located in the capital city of Tanzania (i.e. Dar es Salaam). The population of women students for MUHAS and UDSM were 1,411 and 4,355, respectively. Dar es Salaam University comprises a number of study programmes ranging from pure science to social science. A total of 281 students were randomly selected from the participating institutions proportion to size of women population of the participating institutions. The final sample size was estimated to give the study a power of 80%. However, the power of the study was dropped to 76% because some students did not respond to the questionnaire.The outcome variables included knowledge about contraception, sexual behaviour and contraceptive use. The independent variables were age of the participant, education level, religion, marital status, degree program and source of information (Figure 2).

Sexual behaviour was defined as engagement in sexual activities that may result into unintended health outcomes such as pregnancy and abortion. Knowledge was defined as the state of knowing types of contraceptives.

### Data collection

Prior to data collection, the questionnaire was pretested to 50 female students from another University who did not participate in the study. After pre-testing, the original questionnaire was then modified to ensure that all issues which required amendment were addressed to suit the objectives set. A pretested self-administered questionnaire with closed and open ended questions was administered to 281 students who consented to participate in the study from the studied institutions by proportionate sampling. Of these, 253 responded to the questionnaire. This makes a response rate of 90%.

The University authorities and students were approached, informed consent were obtained from students in the participating institution. The questionnaire consisted of three parts; questions in the first part contained information on the demographic characteristics of the participants. The second part, aimed to assess participants knowledge about contraceptive and use. The third part assessed participant’s sexual experience and contraceptive usage. Specific questions were asked regarding age at sexual debut, source of information on contraceptive, known contraceptive type, ever used and current use of contraceptive. Since the topic under study was sensitive, the investigator first introduced the research topic to students including objectives of the study. The questionnaires were then distributed to the students who consented to participate in the study in the class during the break time. Each participant filled out the questionnaire in private within 15 minutes. Then all questionnaires were collected by the investigator.

### Ethical considerations

Ethical approval was obtained from Institutional Review Board of University of Dar es Salaam and Muhimbili University. The informed consent was obtained from each student who participated in the study. The confidentiality was ensured using anonymous questionnaire where no name of the respondent was used. Participants were informed about the right to withdraw from the study at any time without any effect in accessing their health care.

### Statistical methods

Data was analyzed using Statistical Package for Social Science (SPSS) for Windows version 17.0. Descriptive statistical analysis was used to describe participants’ socio-demographic characteristics, sexual behaviours, knowledge and use of contraception. Student’s t-test was used to compare means between groups for normally distributed continuous variables. The chi square test was used to determine the association between socio-demographic variables, sexual behaviours and contraception usage as categorical variables. A P-value of less than 0.05 was considered as statistically significant. In this manuscript we confirm that our research has adhered to the STROBE guidelines.

## Results

### Socio-demographic characteristics of study participants

A total of 253 students were studied with a response rate of 90% (253/281). Twenty eight students (10%) did not return the questionnaire. As it is shown in Table 1, majority of the participants 172 (68%) were aged 19–23 years with a mean (SD) age of 22.9 (2.4) years. The majority of the respondent 226 (89.3%) were unmarried. One hundred and five (41.5%) were Catholics, 99 (39.1%) were protestants, the remaining 49 (19.4%) were Muslims. The majority 202 (79.8%) were pursuing degree in arts and humanities, 31 (12.3%) were pursuing science degree programs while the rest 20 (7.9%) were pursuing medicine and life science degree programs.

**Table 1:** Sociodemographic characteristics of the participants (N = 253)

**Characteristics**	**Frequency**	**%**
**Age of participants (years) +**	23 (2.4)	
**Age group (years)**		
19-24	172	68.0
25-30	76	30.0
31-37	5	2.0
**Marital status**		
Single	226	89.3
Married	27	10.7
**Religion**		
Catholic	105	41.5
Protestant	99	39.1
Muslim	49	19.4
**Degree program (faculty)**		
Science	31	12.3
Arts and humanities	202	79.8
Medicine and life science	20	7.9
**Year of study**		
First year	53	20.9
Second year	82	32.4
Third year	90	35.6
Fourth year	21	8.3
Fifth year	7	2.8

**+**Mean (Standard Deviation).

### Reproductive and sexual health characteristics and contraceptive use

The relationship between sexual behaviour and contraceptive use is shown in Table 2. Majority 178 (70.4%) of the respondents have had sexual intercourse, 133 (74.7%) had first sex at the age of 19–24 years. The mean (SD) age at sexual debut was 20 (2.4) years, with a range of 13 to 37 years. More than one-third of the respondents started sexual activity when they were in secondary school (34.4%) or university (35%). Forty (15.8%) of the respondents have had pregnancies. Of these, 11 (27%) have had unwanted pregnancies among which 54.6% have had induced abortions.

**Table 2:** Sexual behaviour and contraceptives usage among female students (N = 253)

**Characteristics**	**Frequency (n)**	**%**
**Overall contraceptive usage (ever used)**	148	58.5
**Overall contraceptive usage (current users)**	105	41.5
**Ever had sex**		
Yes	178	70.4
No	75	29.6
***Sexually active participants (N = 178)***		
**Age at first sex (years) +**	20 (2.3)	
**Age group at first sex (years)**		
13-18	40	22.5
19-24	133	74.7
25-30	5	2.8
**Ever used contraception**		
Yes	148	83.1
No	30	16.9
**Current use of contraception**		
Yes	105	59.0
No	73	41.0
Type of contraception used (ever used)*		
Condom	121	56.0
Periodic abstinence	38	17.6
Withdrawal	35	16.2
Pills	16	7.4
Others	6	2.8
Type (current users)*		
Condom	88	60.3
Withdrawal	24	16.4
Periodic abstinence	23	15.8
Pills	6	4.1
Norplant	3	2.1
Injection	1	0.7
Diaphragm	1	0.7

*A respondent could name more than one method, thus the above do not add to total study group of 178 for respondents who are sexually active, **+**Mean (Standard Deviation).

More than half of the participants 148 (58.5%) have ever used any contraceptives while 105 (41.5%**)** were current users. Among the users the most common contraceptive methods were condoms 121 (56.0%), periodic abstinence 38 (17.6%) and withdrawal 35 (16.2%). The periodic abstinence was the commonest contraceptive method used among unmarried students (73.7%), while pills were the method of choice among married participants (66.7%).

### Knowledge about contraception

All respondents were aware of the contraceptives. Majority 221 (86.3%) had ever heard about condoms, 153 (16.8%) had ever heard about pills and 150 (16.5%) had ever heard injectable contraceptive method. The most common sources of information about contraception were friends/relatives (39.5%), radio (36%) and school (24%). The knowledge about contraception varied by age, where respondents aged between 25–30 years had more knowledge about contraceptives methods as compared to other age groups. As expected, students pursuing medicine and life science had higher knowledge on different contraceptive methods than other disciplines (Table 3).

**Table 3:** Contraceptives Knowledge among participants (N = 253)

**Characteristics**	**Frequency**	**%**
**Ever heard about contraceptive**		
Yes	253	100
**Source of information about contraceptives**		
Friends	100	39.5
Radio	91	36.0
Family	33	13.0
Schools	81	32.0
**Modern contraceptive ever heard**		
Pills	153	16.8
Injection	150	16.5
Condom	221	24.3
IUCD	25	2.7
Diaphragm	67	7.4
Spermicidal	41	4.5
Norplant/Implants	2	0.2
**Traditional methods ever heard**		
Periodic abstinence	122	13.4
Withdrawal	116	12.7
Lactation amenorrhea	13	1.4

### Factors associated with contraceptive use

Table 4 describes the factors associated with contraceptive use. Being married and age at first sex of 23 years or more were significantly associated with higher ever use rate of contraception (χ^2^ = 6.58, P = 0.001 and χ^2^ = 14.9 P = <0.001) respectively. On the other hand, ever been pregnancy and unwanted pregnancy were associated with lower ever use of contraception (χ^2^ = 30.9, P = <0.001, and χ^2^ = 10.6, P = <0.001, respectively). Regarding the current usage of contraception, age at first sex of 23 years or more and ever had sex and were significantly related with current use of contraception ( **χ**^**2**^ **=** 4.0 P = 0.03, and **χ**^**2**^ **=** 3.8 P = 0.04, respectively). Consequently, women who had ever been pregnancy and those who experienced unwanted pregnancy had lower current contraception usage (27.5 vs 44.1%) and (27.3 vs 42.1%) respective. But these difference did not reach statistical significant. Fear of pregnancy (49.2%) and fear of contracting HIV/AIDS (17.2%) were the main reasons mentioned to influence the utilization of contraception. However, due to small sample in some groups, it was difficult to perform logistic regression analysis which we believe it could have given the strength of associations for the observed relationships.

**Table 4:** Factors associated with contraceptive use among female students (N = 253)

**Characteristics**	**Ever used contraception**		**Current use contraception**	
	**n (%)**			
	**Yes**	**No**	**Yes**	**No**
**Demographic**				
Marital status				
Single	126 (55.8)	100	91 (40.3)	135
Married	22 (81.5)	5	14 (37.8)	23
Statistical tests	*χ*^2^ **=** 6.6, p = 0.01*		*χ*^2^ = 0.78 p = 0.25	
Age at first sex				
<23	58 (46.4)	67	44 (35.2)	81
≥23	90 (70.3)	38	61 (47.7)	67
Statistical tests	*χ*^2^ = 14.9 p <0.001		*χ*^2^ = 4.0 4 p = 0.03	
**Sexual behaviour**				
Ever had sex				
Yes	108 (60.7)	80	85 (47.8)	93
No	40 (53.3)	35	20 (33.3)	40
Statistical tests	*χ*^2^ **=** 0.37 p = 0.58		*χ*^2^ = 3.8 p = 0.04	
Ever been pregnant				
Yes	7 (17.5)	33	11 (27.5)	19
No	141 (66.2)	72	94 (44.1)	119
Statistical tests	*χ*^2^ = 30.9 p <0.001		*χ*^2^ = 0.59 p =0.56	
Unwanted pregnant				
Yes	1 (9.1)	10	3(27.3)	8
No	147 (60.7)	95	102 (42.1)	140
Statistical tests	*χ*^2^ = 11.6 p < 0.001		*χ*^2^ = 0.96, p = 0.37	

*Fishers exact test p value.

## Discussion

Although majority of the respondents in this study had knowledge of contraception, but we found that the utilization of contraception is still low. Majority of the students were sexually active and started sexual activity at young age. We also found that marital status, age at first sex, ever had sex, ever been pregnancy and unwanted pregnancy were independent associated with use of contraception.

Knowledge about fertility control is an important step towards access to and use of an appropriate contraceptive methods in a timely and effective manner [9]. In the present study all the respondents were aware about contraceptive. Our findings are consistent with previous studies in the region [18]. We however, found there was a difference between participants’ contraceptive knowledge and sexual behaviour, where 70.4% of the participants engaged in sexual activity while only 41.5% reported currently using contraception. Similar trend was reported in another study in Nigeria [15]. Condom was the most commonly known contraceptive and more frequently used while other methods like intra uterine device, lactation amenorrhea and Norplant were rarely mentioned. This could be explained by education campaigns and extensive social marketing of condoms in response to HIV epidemic which has also been reported elsewhere [20,21]. This underscores the need of adopting similar approach in promoting other contraceptive methods in order to increase its use.

The success of any family planning programme activities is determined by the level of current use of contraceptives [9]. In this study, the contraceptive ever use rate was 58.5% while 41.5% of the respondents were currently using contraceptive methods of any type with condom being the commonest method used. The observed contraceptive use rate in our study was high compared with 14.5% that was reported by Byamugisha et al. among university students in Uganda [11]. The difference between contraceptive usage and sexual risk behaviour exposes high proportion of women at risk of unintended pregnancy and other sexual transmitted infections as majority of the participants in the present study were not using contraception. Condom, withdrawal and periodic abstinence methods were the most popular methods ever used or currently being used by respondents. A very similar pattern on the choice of contraceptive method was reported among Nigerian undergraduate students [15]. In the present study, the main reason for using contraception was to avoid pregnancy before graduation (49.2%) while only 17.2% used contraceptive because they feared contracting HIV/AIDS. Similar finding was reported by a previous study in Madagascar [10]. However, the reasons for not using other contraceptive methods remain unclear which requires further studies.

The present study found that about 38.6% of the students had been using contraceptive between 1–2 years. This figure is slightly higher compared with 21.6% reported by a Nigerian investigators [18]. This difference could be attributed to age distribution or sample size differences between the studied populations. In addition, the difference in the exposure duration, and access to contraceptive information between two countries could attribute to the observed difference in contraceptive use.

Our study has showed that the mean age at sexual debut is 20 (2.3) years. This finding corresponds to the study done among Madagascar university students which reported the mean age of 19 years [10]. In this study, we found that about 70.4% of the study participants reported to have ever had sexual intercourse. This proportion is slightly lower as compared to 79% that was reported among Ethiopian students [22]. Given the relatively high degree of sexual activity and limited use of effective contraceptive methods by adolescents, these students are at great risk of acquiring sexual transmitted diseases including HIV etc., unwanted pregnancy and illegal abortions. This was the case in our study where we found that 11(26.8%) women had unwanted and 6 (54.6%) them terminate their pregnancies. In contrast, a study among female Nigerian undergraduate students reported the abortion rate of 34% [19]. The likely explanation for this difference could be due to difference in sample size and social cultural differences between two studies.

Apart from the methods requiring direct contact with the health workers; pharmacy and shops were commonest source of modern contraceptive methods. Our results is in agreement with previous study among Nigerian undergraduates [15], where authors reported pharmacy was among the commonest sources of contraceptive services. Despite the fact that the majority of the students in the current study reported to obtain contraceptive services from the pharmacies and shops, (75.2%) would prefer to get contraceptive services from the university health centre and (14.3%) from the halls of the residence. Probably this could be due to an easy access to these facilities. The findings also underscores the need to provide contraceptive information’s in shops and pharmacies possibly via advertisement, posters and brochures in addition to providing contraceptive services in the university’s health clinics.

### Strengths and weaknesses of the study

This study has some limitations which need to be considered while interpreting our results. First, the study was carried out only in two universities which might not be representative of Tanzania university female students, thus results may not be generalizable to other universities in Tanzania. Second, due to the nature of the study which involved sensitivity matter and self-reporting, information bias could be introduced which may have affected reliability of the results. Third, since this was a cross-sectional study design, it is difficult to assess the cause and effect relationship. Fourth, we were not able to study the characteristics of the students who declined to participate. These students might have different characteristics from those who participated in the study. But the proportion of non-responder was very small to change affect our results.

This study has provided important baseline information regarding the pattern of contraceptive use among female university students in in the studied institutions. In addition, our findings provide insight to program managers on how and to whom educational message on contraception should be targeted. Future research should explore on the barrier for utilization of contraceptive in the studied and recruit more participants.

## Conclusions

This study showed that the knowledge about contraception among the female students was high. Majority of the students were sexually active and started sexual activity at earlier age. However, the rate of contraception use is still low. The low contraceptive usage suggests the need for sexual and reproductive health education program to promote use of contraceptive services in the study settings. The reproductive health education programs should include the importance of using dual contraceptive methods as a means to prevent HIV transmission as well as prevent pregnancy. Additional research studies are needed to understand the barriers for contraceptive use in particular type of contraceptives used and reasons for not utilizing services provided in health facilities within university campuses. Future studies also need to use rigorous recruitment approaches to enhance high participation rate.

## Competing interests

The authors declare that they have no competing interests.

## Authors' contributions

MJS conceived of the study idea, designed the study, carried out the statistical analyses, interpretation of the results, and drafted the manuscript. MM participated in the design of the study, reviewed the manuscript. MJM provided technical support for statistical analysis, interpreting results, drafted the manuscript and reviewed it for intellectual content. JO reviewed the manuscript for intellectual content. All authors read and approved the final manuscript.

## Pre-publication history

The pre-publication history for this paper can be accessed here:

http://www.biomedcentral.com/1472-6874/14/94/prepub
